# The 2021 NICE guidelines for assessment and management of chronic pain: A cross-sectional study mapping against a sample of 1,000* in the community

**DOI:** 10.1177/20494637221083837

**Published:** 2022-04-05

**Authors:** Zoe Zambelli, Elizabeth J Halstead, Ray Iles, Antonio R Fidalgo, Dagmara Dimitriou

**Affiliations:** 1 Sleep Education and Research Laboratory, Psychology and Human Development, 4919 University College London-Institute of Education, London, UK; 2 The National Institute for Stress, Anxiety, Depression and Behavioural Change (NISAD), Helsingborg, Sweden; 3 Department of Psychology, 4917 University of East London, London, UK

**Keywords:** Pain management, healthcare guidelines, mental health, primary care

## Abstract

**Objectives:**

To characterise the prevailing pharmacological and non-pharmacological pain management strategies among adults with chronic pain, comparing these against the newly published NICE guidelines NG-193, and examine these pre-NG-193 pain management strategies in relation to pain severity, pain interference, sleep quality and mental health outcomes.

**Design:**

This study was conducted using a cross-sectional online survey study design.

**Setting:**

This study was conducted on a community-dwelling cohort.

**Participants:**

Adults aged 18+, living in the UK, with diagnosis of chronic pain by a health care professional.

**Main outcome measures:**

Primary outcomes were characterisation of the pain management strategies utilised. Secondary outcomes were related to pain severity, pain interference, sleep quality, depression and anxiety via validated self-report measures.

**Results:**

Several strategies were employed by respondents to manage their chronic pain condition including physical therapy, exercise, psychological therapy and pharmacological therapy. The data also indicated a high level of joint-care planning among patients and their clinicians. Some group differences were found in relation to pain, sleep and mental health outcomes.

**Conclusion:**

This study set a comparative starting baseline to which the efficacy of the NG-193 may be compared in future years. There is evidence that NICE recommendations are being followed for the management of chronic primary pain conditions; however, pharmacological use of opioid drugs is still reported by 47%. Despite the confirmed evidence in this study of small efficacy of chronic pain by pharmacological agent, the reduction in the use of pain relief medications be it over the counter medications or prescription opioids, as recommended by NG-193, may be slow to be adopted. The data suggest that more care provision is needed to meet the recommendations around pharmacological management and review.

## Introduction

In the UK, circa one third of the population is impacted by chronic pain (CP), with up to 15% reporting this to be severe enough to impair daytime functioning and be disabling.^1^ In 2021, new guidelines for assessment of chronic secondary pain and chronic primary pain (CPP) and management of CPP in over 16-year-olds have been published by the National Institute of Health and Care (NICE guidelines NG-193, 2021). It is the first time that CPP has been classified as a condition in its own right by the NICE, aligning with the International Classification of Diseases 11th Edition (ICD-11).

**Figure 1.**
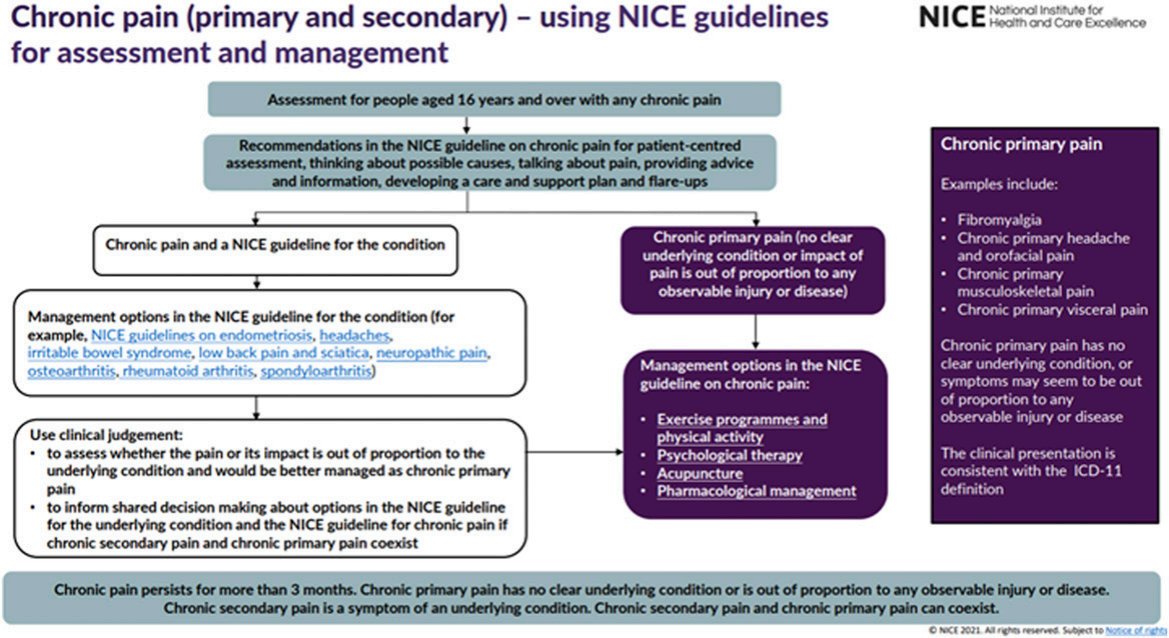
National Institute of Health and Care NG-193 visual summary

The assessment component within the NG-193 guidelines emphasises a person-centred approach to assessing an individual presenting with chronic pain. This includes offering tailored advice to ultimately reach a joint-care plan incorporating the clinician’s expertise and patient’s preferred approach to treatment. Secondly, the NG-193 guidelines recommend patient-centred care with an emphasis on non-pharmacological management of pain which include (1) Exercise programmes and physical activity, (2) Psychological therapy and (3) Acupuncture. Significantly, NICE recommend pharmacotherapies such as paracetamol, non-steroidal anti-inflammatory drugs (NSAIDs), benzodiazepines, antiepileptic drugs and opioids *are not* initiated with CPP patients. Figure 1 displays an infographic published by NICE which summarises the guidelines (NICE, 2021).

The NG-193 guidelines have been met with controversy. Concerns centre around disparate issues such as : the over-simplification of CPP as a condition,^2^ the recommendations around pharmacological treatment for CPP^3^ and pain education guidance.^4^ Despite these concerns, the guidelines offer a way to facilitate evidence-based practice for individuals with chronic primary pain for the first time, and do so following a vast evidence review.

The NG-193 guidelines are the first iteration of guidance for assessment and management of CPP. It is therefore important to begin contextualising current practice management and support health care professionals to implement these. Our current study offers a baseline of current practice management from real-world data whilst identifying potential barriers to implementation and recommendations on how these may be overcome. The NICE guidelines recognise the importance of good mental health and sleep practices for CPP populations. This is highlighted by (1) emphasis of discussing lifestyle, psychological and physical health factors when assessing patients, (2) the recommendation for psychological therapies to improve psychological distress and sleep outcomes in this group and (3) forming part of the rationale for recommending exercise which improves quality of life outcomes. Therefore, the present study also aims to examine the observed associations between a variety of adopted pain management strategies on pain interference, pain severity, sleep quality, depression and anxiety.

Two objectives were identified:

1. Characterise pain management strategies among adults with chronic pain with particular attention to prevalence of pharmacological treatment, psychological therapies and physical activity in order to capture a baseline of current practice management which may be compared in future to standards set out in NG-193.
2. Examine the associations of pain management strategies in relation to pain, sleep and mental health outcomes in recognition of their impact on the experience of chronic pain.

## Methods

### Design

A cross-sectional survey was conducted using Qualtrics XM (www.qualtrics.com), survey management software, between February and March 2020. Participants provided informed consent prior to completing the survey and did not receive compensation. Inclusion criteria were adults aged 18 years or older, with diagnosis of chronic pain by a healthcare professional. Exclusion criteria were diagnoses of cancer-related chronic pain and pain duration of <3 months.

### Ethics approval and consent

Ethical approval was granted by University College London, Institute of Education Ethics Committee. Written informed consent was gained prior to survey completion by the participants.

### Participants

A total of 1234 participants fully or partially completed the survey relating to their pain management and other health outcomes. Participants were recruited through a social media campaign and in collaboration with UK pain charities. Individuals with missing data pertaining to their pain condition were excluded. There were 1,176 fully completed responses including demographic characteristics and outcome measures within this study. Most participants identified as female (88%), had a mean age of 43 years (*SD* = 13.5) and were of white ethnic origin (93%). Participants reported Chronic Widespread Pain (CWP; 34%), Musculoskeletal (MSK; 35%), Headache and Orofacial (12%), Neuropathic (16%) and Visceral pain conditions (3%). More than half the sample reported pain duration >10 years (57%). Participant characteristics are reported in Table 1.

**Table 1.** Participant characteristics.

Variable		N	%
Age^a^		43	13.5
Sex	Female	1039	88
	Male	137	12
Ethnicity			
	White-any background	1097	93
	Black/Black British	6	1
	Asian/Asian British	11	1
	Mixed	33	3
	Other	29	2
Chronic pain condition			
	Chronic widespread pain	396	34
	Musculoskeletal	414	35
	Headache and Orofacial	142	12
	Neuropathic	187	16
	Visceral	37	3
Pain duration			
	≤1 year	25	2
	>1–≤3 years	105	9
	>3–≤ 5 years	111	9
	>5–≤10 years	252	22
	>10 years	671	57
	Unsure	12	1
Co-morbid physical health condition			
	Yes	885	75
	No	291	25
Co-morbid mental health condition			
	Yes	628	53
	No	548	47
BPI pain severity score^a^		5.5	1.83
BPI pain interference score^a^		6.37	2.22
Pittsburgh Sleep Quality Index sleep quality score^a^		13.84	3.84
HADS-D depression score^a^		9.1	4.46
HADS-A anxiety score^a^		10	4.86

BPI: Brief Pain Inventory; HADS: Hospital Anxiety and Depression Scale

^a^denotes variables which are on a continuous scale and therefore display mean (M) and standard deviation (SD) values.

### Measures

Participants completed background questions in addition to three validated questionnaires to measure pain interference, pain severity, sleep quality, anxiety and depression.

### Demographic Information

Participants answered questions relating to their primary chronic pain condition, pain duration, time since diagnosis, healthcare professional who diagnosed condition, type and number of pain management strategies currently employed, types and number of pain medications used and shared care planning with HCP. Free text responses were collected regarding types of pain management strategies adopted and pain medication used.

Demographic indicators included, age, gender, ethnicity, education attainment and employment status (see *Participants)*.

### Pain

The Brief Pain Inventory (BPI-SF) is a widely used self-report measure for clinical pain.^5^ The BPI includes two subscales which rate severity of pain and the degree to which pain interferes with common dimensions of feeling and function in the past 24 h. These are referred as (1) pain severity and (2) pain interference. Subscales range from 0–10 with higher scores indicating higher levels of severity and interference. The BPI has shown good internal consistency across both subscales; α = 0.85.^6^

### Sleep quality

The Pittsburgh Sleep Quality Index (PSQI) consists of 24 items.^7^ The scale comprises seven components which measure (1) subjective sleep quality, (2) sleep latency, (3) sleep duration, (4) sleep efficiency, (5) sleep disturbance, (6) daytime dysfunction and (7) sleep medication over the past month. Each component generates a score from 0–3 where higher scores indicate poorer sleep outcomes. A sum of seven component scores is used to generate a global PSQI score ranging from 0–21. A global score above five indicates poor sleep quality. The PSQI has previously been validated among chronic pain populations and exhibits good reliability (α = 0.7).^8^

### Anxiety and depression

The Hospital Anxiety and Depression Scale (HADS) is a 14-item validated measure designed to measure anxiety and depression symptoms during the past week.^9^ It comprises of two subscales assessing (1) anxiety and (2) depression. Items are rated on a four-point Likert scale. Scores for each item are summed for each subscale, scores above eight suggest anxiety and depression are present with thresholds described as 0–8 = no symptoms, 8–10 = mild symptoms, 11–14 = moderate symptoms, 15–21 = severe symptoms.^10,11^ The HADS has previously been validated in chronic pain populations, with demonstration of good internal reliability.^12^

### Analysis plan

Descriptive statistics were used to characterise the sample (Table 1). Continuous data were presented as a mean and standard deviation. Nominal data were presented as total number of participants (and percentage) that featured in each corresponding category. Table 2 addressed aim 1 which we used to summarise diagnosis, time since diagnosis, pain management strategy, type of medication and number of medications taken as nominal data. Treatment plan was presented as a categorical (yes/no) variable.

**Table 2.** Pain management characterisation among community-dwelling adults with chronic pain.

Variable		N	%
Diagnosed by			
	GP (General Practitioner)	96	8
	Speciality doctor	1026	87
	Allied health professional	25	2
	Other	29	3
Time since diagnosis			
	≤1 year	124	11
	>1–≤3 years	199	17
	>3–≤5 years	168	14
	>5–≤10 years	280	24
	>10 years	392	33
	Unsure	13	1
Pain management strategy			
	Medication (prescribed and over the counter)	1093	93
	Medication (prescribed only)	903	77
	Physical activity	274	23
	Physical therapy	425	36
	Psychological therapy	170	15
	Surgery waitlist	72	6
	Weight management	103	9
	None	112	10
	Other	244	21
Types of medication			
	Anticonvulsants	158	13
	Antidepressants	394	34
	Compound painkiller	251	21
	Corticosteroids	111	9
	Non-steroidal anti-inflammatory drugs	487	41
	Opioids	547	47
	Paracetamol	548	47
	Triptans	52	4
	Other	162	14
No. of medications			
	None	83	7
	1	253	22
	2	270	23
	3	274	23
	4 or more	296	25
Treatment plan prescribed by a healthcare professional			
	Yes	1017	89
	No	126	11

**questions which allowed multiple select choices.

Before embarking on statistical analyses, pain interference, pain severity, sleep quality, depression and anxiety data were tested for normality using skewness and kurtosis values between +1 and −1. Tables 3–5 address aim 2, where we conducted seven general linear models of multivariate analysis of covariance (MANCOVA) and tested the difference between pain interference, pain severity, sleep quality, depression and anxiety and several pain management strategies. In each model, we used age, sex and pain duration as possible covariates. Pillai’s Trace was used to report on the model’s significance due to unequal group samples. A *p*-value of <.05 was considered significant. Following a significant result, pairwise comparisons using Bonferroni corrections were used to examine between-group differences.

**Table 3.** Between subject differences in pain, sleep quality and mental health based on utilisation of non-steroidal anti-inflammatory drugs, opioids and antidepressant medications.

**Variable**	**Non-steroidal anti-inflammatory drugs (M, SD)**				
**F(5, 1167) = 1.93, p = .083, Pillai’s Trace = 0.008, *n*2 = 0.008**					
**Variable**	**Opioids (M, SD)**				
**F(5, 1167) = 23.83, p = <.001, Pillai’s Trace = 0.093, *n*2 = 0.093**					
	**Yes (*n* = 547)**	**No (*n* = 629)**	***F(5, 1167)***	***p-*value**	***n*** ^***2***^
Pain interference	6.87 (1.96)	5.95 (2.35)	48.801	<.001	0.04
Pain severity	5.96 (1.65)	5.1 (1.9)	62.016	<.001	0.05
Sleep quality	14.88 (4.45)	12.95 (3.94)	75.12	<.001	0.06
Depression	9.95 (4.47)	8.35 (4.32)	37.607	<.001	0.031
Anxiety	10.21 (4.92)	9.76 (4.8)	2.528	.112	0.002
**Variable**	**Antidepressants (M, SD)**				
**F(5, 1167) = 8.94, p = <0.001, Pillai’s Trace = 0.037, *n*2 = 0.037**					
	**Yes (*n* = 394)**	**No (*n* = 782)**	***F(5, 1167)***	***p-*value**	***n*** ^***2***^
Pain interference	6.76 (2.09)	6.18 (2.26)	17.217	<.001	0.014
Pain severity	5.74 (1.72)	5.37 (1.88)	9.62	<.001	0.008
Sleep quality	14.66 (3.62)	13.43 (3.88)	27.094	<.001	0.023
Depression	10.09 (4.47)	8.6 (4.38)	29.507	<.001	0.025
Anxiety	10.96 (4.86)	9.47 (4.78)	24.549	<.001	0.021

**Table 4.** Between subject differences in pain, sleep quality and mental health based on number of medications taken for pain management.

**Variable**	**Number of pain medications taken**							
**F(20, 4668) = 2.72, p = <.001, Pillai’s Trace = 0.047, *n*2 = 0.012**								
	**0 (*n* = 83)**	**1 (*n* = 253)**	**2 (*n* = 270)**	**3 (*n* = 274)**	**4 or more (*n* = 296)**	***F(20, 4668)***	***p-*value**	***n* ^*2*^**
Pain interference	5.8 (2.57)	6.07 (2.47)	6.22 (2.26)	6.49 (2)	6.83 (1.96)	6.14	<0.001	0.02
Pain severity	5.39 (2.14)	5.17 (2.02)	5.43 (1.86)	5.48 (1.6)	5.87 (1.72)	5.27	<0.001	0.02
Sleep quality	12.72 (3.72)	13.28 (4.19)	13.59 (3.74)	13.95 (3.86)	14.78 (3.44)	8.56	<0.001	0.03
Depression	8.56 (4.62)	8.46 (4.48)	8.84 (4.18)	9.26 (4.48)	9.88 (4.32)	4.51	0.001	0.02
Anxiety	9.84 (5.18)	9.3 (4.8)	9.43 (4.59)	10 (4.94)	11.04 (4.82)	4.31	0.002	0.02

**Table 5.** Between subject differences in pain, sleep quality and mental health based on psychological therapy, physical therapy and exercise.

**Variable**	**Physical therapy (M, SD)**				
**F(5, 1167) = 2.38, p = 0.1, Pillai’s Trace = 0.01, *n*2 = 0.01**					
**Variable**	**Psychological therapy (M, SD)**				
**F(5, 1167) = 2.38, p = 0.037, Pillai’s Trace = 0.01, *n*2 = 0.01**					
	**Yes (*n* = 170)**	**No (*n* = 1006)**	***F(5, 1167)***	***p-*value**	***n*** ^***2***^
Pain interference	6.84 (2.07)	6.3 (2.24)	8.45	.004	0.007
Pain severity	5.78 (1.69)	5.45 (1.86)	5.01	.025	0.004
Sleep quality	14.55 (3.75)	13.72 (3.84)	7.51	.006	0.006
Depression	9.67 (4.26)	9 (4.49)	3.46	.063	0.003
Anxiety	10.88 (4.79)	9.81 (4.86)	4.11	.043	0.004
**Variable**	**Exercise (M, SD)**				
**F(5, 1167) = 7.46, p = <0.001, Pillai’s Trace = 0.031, *n*2 = 0.031**					
	**Yes (*n* = 274)**	**No (*n* = 902)**	***F(5, 1167)***	***p-*value**	***n*** ^***2***^
Pain interference	5.91 (2.16)	6.52 (2.22)	17.86	<.001	0.015
Pain severity	5.22 (1.67)	5.58 (1.88)	9.60	.002	0.008
Sleep quality	12.91 (3.99)	14.13 (3.75)	22.77	<.001	0.019
Depression	7.94 (4.15)	9.45 (4.5)	23.98	<.001	0.02
Anxiety	9.36 (4.46)	10.15 (4.96)	4.59	.032	0.004

All data were analysed using SPSS v.26 (IBM statistics).

### Patient and public involvement

A small group of participants piloted a draft questionnaire before the official launch. Over 20 UK pain charities were involved in dissemination of the questionnaire link to aid recruitment.

## Results

Objective 1: Characterising pain management among a community-dwelling sample of adults with chronic pain

Table 2 displays the characterisation questions regarding participants’ chronic pain and management (*n* = 1176). Most of the sample had been diagnosed by a speciality doctor such as a rheumatologist (*n* = 1026, 87%), in comparison to those who reported a diagnosis via GP (*n* = 96, 8%) or allied health professional (AHP) (*n* = 25, 2%). One third of participants had been diagnosed over 10 years prior (*n* = 392, 33%), and almost one quarter between five and 10 years prior (*n* = 280, 24%).

Participants selected categories which described strategies they were currently employing in addition to an ‘other’ and free-text option. Most selected were use of medication (prescribed) by 77% of the sample (*n* = 903) and medication (prescribed and over the counter), reported by 93% of participants (*n* = 1093). Over one third were engaging in physical or occupational therapy (*n* = 425, 36%) and nearly one quarter in general physical activity (*n* = 274, 23%). 15% of the sample reported having started a course of psychological therapy (*n* = 170). Almost 10% reported using weight management as a strategy, where appropriate (*n* = 103). A smaller percentage reported being on a surgery waitlist related to their chronic pain condition (*n* = 72, 6%). Finally, one in 10 individuals reported not actively managing their pain condition (*n* = 112, 10%). One fifth (21%) indicated using ‘other’ strategies not listed which, upon review of the free-text answers, common strategies were ‘marijuana or CBD remedies’, ‘relaxation and mediation’ and ‘acupuncture’.

The survey included a section about pain medication. Participants were asked to select medication(s) being taken on a regular schedule. Categories of commonly prescribed and OTC medications for chronic pain conditions were available. Participants could select an ‘other’ free-text option. Only 7% did not report use of medication (*n* = 83). Medications available OTC were commonly used: paracetamol was most selected, with nearly half the sample reporting its use at present (*n* = 548, 47%) and non-steroidal anti-inflammatory drugs were also used by over 40% (*n* = 487). Opioid use was reported by 47% of the sample and were the second most selected category (*n* = 547). Antidepressant medication use was reported by one third of the sample (*n* = 394, 34%). One fifth selected compound analgesics (e.g. co-codamol; *n* = 251, 21%). Other medications were triptans (usually prescribed for migraine; *n* = 52, 4%), anticonvulsants (e.g. gabapentin prescribed for neuropathic pain; *n* = 158, 13%) and corticosteroids (e.g. steroid injections; *n* = 111, 9%). Of those who selected ‘other’ as an option (*n* = 162, 14%), common answers were ‘CBD oil’, ‘medical marijuana’ and ‘muscle relaxants’. Regarding number of medications used, one fifth reported taking one medication type (22%), just over one fifth were taking either two (*n* = 253, 22%) or three types (*n* = 270, 23%). One quarter reported using four or more medications as part of their current pain management plan (*n* = 296, 25%).

Finally, the majority of the sample reported that their pain management plan had been prescribed by an HCP (*n* = 1017, 89%).

Objective 2: Examining the associations of pain management strategies in relation to pain, sleep and mental health outcomes

We examined three types of pain management strategies and their respective associations with pain severity, pain interference, sleep quality, depression and anxiety. Pain strategies chosen to be examined were linked to the NICE NG-193 guidelines: (1) medication, (2) psychological therapy and (3) physical activity.

Table 3 displays MANCOVA analyses which examined the impact of three different medication types on the pain, sleep and mental health outcomes, when controlling for duration of pain, age and gender.

There was no statistically significant difference between utilisation of NSAID medication on the combined dependent variables after controlling for pain duration, sex and gender, *F*(5, 1167) = 1.93, *p =* 0.083, Pillai’s Trace = 0.008. Based on this, there was no further examination of the results.

A statistically significant difference between utilisation of opioid medication was found on the combined dependent variables after controlling for pain duration, age and gender, *F(5, 1167)* = 23.83*, p = <.001,* Pillai’s Trace = 0.093, *n*^*2*^ = 0.093, representing a medium to large effect size.^13^ There were significant between-subject differences for pain interference, pain severity, sleep quality and depression scores. Across these domains, individuals who reported taking opioid medication reported poorer outcomes related to pain, sleep quality and depression, than individuals who did not report taking opioid medication. There were no significant differences in anxiety scores across the two groups (*p = .11).*

A statistically significant difference between utilisation of antidepressant medication was found on the combined dependent variables after controlling for pain duration, age and gender, *F(5, 1167)* = 8.94*, p = <.001,* Pillai’s Trace = 0.037 *n*^*2*^ = 0.037, representing a small to medium effect size. There were significant between-subject differences for pain interference, pain severity, sleep quality, depression and anxiety scores. Across all domains, individuals who reported taking antidepressant medication reported poorer outcomes related to pain, sleep quality, depression and anxiety than individuals who did not report taking antidepressant medication.

Table 4 displays the results from a MANCOVA used to examine the relationship between number of medications used by respondents to manage their pain, on outcomes related to pain, sleep quality, depression and anxiety. A statistically significant difference between utilisation of antidepressant medication was found on the combined dependent variables after controlling for pain duration, age and gender, *F(20, 4668)* = 2.72*, p = <.001,* Pillai’s Trace = 0.047 *n*^*2*^ = 0.012, representing a small effect size. There were significant between-subject differences for pain interference, pain severity, sleep quality, depression and anxiety scores. Bonferroni pairwise comparisons revealed that individuals taking four or more types of medication (*M* ***=*** 6.83, *SD* = 1.96) had significantly higher pain interference scores than those individuals taking one (*M =* 6.07, *SD =* 2.47), two (*M =* 6.22, *SD =* 2.26), three (*M =*6.49, *SD =* 2) or no types of medication (*M* ***=*** 5.8, *SD =* 2.57). Individuals taking four or more types of medication (*M =* 5.87, *SD =* 1.72) had significantly higher pain severity scores than those individuals taking one (*M =* 5.17, *SD =* 2.02) or two (*M =* 5.43, *SD =* 1.86) types of medication. Individuals taking four or more types of medication (*M =* 14.78, *SD =* 3.44) had significantly poorer sleep quality than those individuals taking one (*M =* 13.28, *SD =* 4.19), two (*M =* 13.59, *SD =* 3.74) or no types of medication (*M =* 12.72, *SD =* 3.72). Individuals taking four or more types of medication (*M =* 9.88, *SD =* 4.32) had significantly higher depression scores than those individuals taking one (*M =* 8.46, *SD =* 4.48) or two (*M =* 8.84, *SD =* 4.18) types of medication. Finally, Individuals taking four or more types of medication (*M =* 11.04, *SD =* 4.82) had significantly higher anxiety scores than those individuals taking one (*M =* 9.3, *SD =* 4.8) or two (*M =* 9.43, *SD =* 4.59) types of medication.

Table 5 displays the results from a MANCOVA used to examine the relationship between psychological therapy, physical therapy and exercise, on outcomes related to pain, sleep quality, depression and anxiety. There was no statistically significant difference between access to physical therapy on the combined dependent variables after controlling for pain duration, age and gender, *F(5, 1167)* = 1.71*, p = 0.13,* Pillai’s Trace = 0.007, *n*^*2*^ = 0.007. Based on this, there was no further examination of the results.

There was a statistically significant difference between access to psychological therapy on the combined dependent variables after controlling for pain duration, age and gender, *F(5, 1167)* = 2.38*, p = .037,* Pillai’s Trace = 0.001, *n*^*2*^ = 0.001, representing a small effect size. There were significant between-subject differences for pain interference, pain severity, sleep quality and anxiety scores. Across these domains, individuals who reported accessing psychological therapy reported poorer outcomes related to pain, sleep quality and anxiety than individuals who did not report accessing psychological therapy. However, there were no significant differences in depression scores across the two groups (*p = .06).*

There was a statistically significant difference between general exercise status on the combined dependent variables after controlling for pain duration, age and gender, *F(5, 1167)* = 7.46*, p = <.001,* Pillai’s Trace = 0.031, *n*^*2*^ = 0.031, representing a small to medium effect size. There were significant between-subject differences for pain interference, pain severity, sleep quality, depression and anxiety scores. Across these domains, individuals who reported adopting physical activity reported better outcomes related to pain, sleep quality, depression and anxiety than individuals who did not report performing physical activity.

## Discussion

This study aimed to characterise pain management among a sample of adults with non-malignant chronic pain conditions, comparing trends against newly published NICE guidelines for management of chronic pain. It also examined associations between pain management strategies and outcomes related to pain, sleep and mental health among this cohort in recognition that these factors often influence the pain experience in addition to being influenced by chronic pain itself.

### Characterising chronic pain management and providing a baseline of current practice management

Our characterisation of pain management revealed several strategies implemented by individuals with chronic pain, some which depend on health services and practitioners, others based on principles of self-management. Firstly, our data illustrated that individuals with chronic pain are creating their management plans with agreement and input of a healthcare professional; a principle adopted within the NG-193 guidelines. This demonstrates a level of shared decision making between clinicians and patients.

Most respondents reported the use of medication as part of their pain management regime which supports data from primary care research.^14,15^ Furthermore, we identified high use of prescribed (e.g. opioids) and OTC medications (e.g. NSAIDs) which is concordant with data from a large survey study across Europe in which use of NSAIDs was reported by 55% of respondents and paracetamol use reported by 43% of respondents.^15^ Research has demonstrated that patient–clinician interaction surrounding medication can be challenging, in particular, instances where clinicians and patients are in disagreement over initiation, tapering or dosage.^16^ Given the NG-193 guidelines recommend a medication review with the intention of reducing and eliminating several medication types for management of CPP, we consider that this may require some time before it is regularly adopted. This is owing to the resource required to carry out these reviews due to high prevalence of medication use, in addition to the significant change from current practice. Finally, there may be additional challenges due to difficult clinician–patient communication with regards to discussions about pain medication which may also impact the implementation of this recommendation.^17,18^

One sixth of participants engaged with psychological therapy. The NG-193 guidelines recommend ACT (acceptance and commitment therapy) and CBT (cognitive behavioural therapy); however, our study did not assess the type of therapy individuals engaged with, the format of delivery or completion rate. Furthermore, data from the present study demonstrate high engagement of supervised and unsupervised exercise, with one third reporting uptake of a structured exercise programme and one quarter reporting incorporating unstructured physical activity which are included within the NG-193 guidance.

### Examining the associations of current pain management strategies and pain, sleep and mental health outcomes

The NG-193 guidelines highlight the burden of chronic pain on several health-related outcomes, recognising external factors which may impact pain, and recommend these be discussed within a holistic approach to care. Our data offer a detailed depiction of pain, sleep and mental health outcomes across this group in correlation to pain management strategies being employed.

The NG-193 guidelines garnered attention for their recommendations around pharmacological treatment. In particular, the recommendation against initiating several classes of medicines used for pain management. Our results indicate that in individuals taking either opioids or antidepressants reported outcomes significantly poorer across several domains, for example, pain, sleep and depression, in comparison to individuals not taking these medication types. Individuals who reported taking four types of medications or more or manage their pain also had significantly poorer outcomes compared to those individuals taking fewer medications. These results may reflect that medication and polypharmacy are utilised among more severe cases of chronic pain. Due to the cross-sectional nature of this study, it is not possible to make inferences about directional relationships. However, our data support the notion that review of medicines is needed within this population to ensure individuals are receiving optimal treatment which take into consideration both risks and benefits of these medication types within a treatment plan. This is supported not only as part of the novel NG-193 guidelines but also as part of previous medicines optimisation strategies published by the NICE.^19^

Furthermore, our data revealed that those utilising psychological therapy had poorer outcomes related to pain, sleep and anxiety than those who were not. Depression scores across these two groups did not differ, which may suggest that the models of therapy used were targeting depression, as is common within the NHS. Evidence has shown that ‘standard clinical cases’ report better clinical outcomes following psychological therapy than ‘complex clinical cases’.^20^ Although there is no set definition for ‘complex clinical cases’, there is a consensus that individuals with more complex needs should be offered high intensity therapies, for example, ACT and CBT to improve outcomes. Drop-out rates for these types of therapies, particularly within IAPT (Improving Access to Psychological Therapies), are high, and completion may be atypical in real-world data.^21^ These results may reflect a higher severity of symptoms for individuals who are referred to these specialist services and suggest a need for increased service provision to replicate the outcomes in published trials.

Finally, analyses revealed significantly better outcomes related to pain, sleep, depression and anxiety for individuals who incorporated exercise as part of their pain management compared to individuals who did not. This supports data which advocate for physical activity as a safe and effective strategy in improving health outcomes for individuals with chronic pain and supports the recommendation for physical activity within the NG-193 guidelines.^22^

### Limitations

This study relied on self-report measures which may misalign with objective measures.^23^ Despite this, self-report measures offer an accessible solution to data gathering and are relied upon within the NHS to capture clinical outcomes, particularly within pain populations.^24^ Secondly, most participants identified as ‘white’ ethnicity and as women; therefore, the study is limited in generalisability to other ethnic and sex groups, future studies should seek to engage more diversity in responses (Fryer et al., 2016). However, we acknowledge data that indicates prevalence rates for chronic pain may be higher in women, compared to men,^1,15,25^ in addition to the underutilisation of healthcare services observed within BAME communities.^26,27^ Finally, this research was conducted on a community sample and therefore offers results from individuals who may be accessing health services beyond those offered by the NHS, who do not follow NICE guidelines.

### Implications and recommendations

1. Assessment guidelines supported within the NG-193 are being implemented and there is evidence of joint care planning occurring among HCPs and patients.
2. The use of structured exercise and promotion of physical activity overall is supported at the core of pain management for chronic pain, offering a viable and safe strategy for management across several conditions.
3. Service provision to access psychological therapies should be increased in order to replicate RCT results and meet the NICE guideline recommendation as a management strategy for CPP. In addition, targeted interventions may need to be adapted for CPP populations.
4. The use of medications and polypharmacy is prevalent among chronic pain populations and may negatively impact aspects of well-being, whilst still aiding with symptom control. Healthcare services will require resource in order to implement NG-193 recommendations to reduce use of medications among this population. Special provision within primary care in particular should be made to review medications.

## Conclusion

The NG-193 guidelines offer the first iteration of treatment recommendations targeted for individuals with CPP. The aim of this paper was to outline commonly used strategies for management of chronic pain among a community sample, many of whom are accessing health services to form their care plans. Our findings indicate that physical therapy is being accessed by a large percentage of this cohort, in addition to using unsupervised physical activity to manage chronic pain. These are strategies recommended at the core of the new NG-193 guidelines and are shown to be safe and effective. Additionally, a small percentage of individuals utilised psychological therapy. Given that self-referral is a valid mechanism for some psychological services within the NHS, the limited uptake may suggest a service provision issue, or lack of awareness of services for individuals with CPP. High dropout rates are also common among these interventions and may explain the smaller percentage completing these interventions. Identifying barriers to uptake in this population may be important in meeting this NICE recommendation. Finally, pharmacotherapy was found to be the most common strategy for pain management among this group. Although unsurprising, the NICE guidelines propose a radical shift to the way medications are used to manage many CPP conditions. Health services will need time and resource to implement the new recommendations which may be met with resistance from patients themselves.

## References

[1] Fayaz A Croft P Langford RM , et al. Prevalence of chronic pain in the UK: a systematic review and meta-analysis of population studies. BMJ Open 2016; 6(6): e010364. 10.1136/bmjopen-2015-010364 PMC4932255 27324708

[2] Eccleston C Aldington D Moore A , et al. Pragmatic but flawed: the NICE guideline on chronic pain. The Lancet 2021; 397(10289): 2029–2031. 10.1016/S0140-6736(21)01058-8 34062133

[3] Kmietowicz Z . Doctors raise concerns about NICE guidelines on chronic primary pain. London, UK: British Medical Journal Publishing Group, 2021. 10.1136/bmj.n942 33837001

[4] Ryan C Walumbe J Moseley GL . Naughty or NICE Chronic pain guidelines: a commentary on pain education guidance. Pain Rehabilitation-The J Physiother Pain Assoc 2021; 2021(50): 1–3.

[5] Cleeland CS Ryan K . Pain assessment: global use of the Brief Pain Inventory. Singapore: Annals, academy of medicine, 1994. 8080219

[6] Tan G Jensen MP Thornby JI , et al, Validation of the Brief Pain Inventory for chronic nonmalignant pain. The J Pain 2004; 5(2): 133–137. 15042521 10.1016/j.jpain.2003.12.005

[7] Buysse D . The pittsburgh sleep quality index (PSQI): a new instrument for psychiatric practice and research. Pittsburgh, PA: Elsevier Scientific Publishers Ireland, 1989. 10.1016/0165-1781(89)90047-4 2748771

[8] Nicassio PM Ormseth SR Custodio MK , et al. Confirmatory factor analysis of the pittsburgh sleep quality index in rheumatoid arthritis patients. Behav Sleep Medicine 2014; 12(1): 1–12. 10.1080/15402002.2012.720315 PMC4285368 23390921

[9] Zigmond AS Snaith RP . The hospital anxiety and depression scale. Acta Psychiatrica Scandinavica 1983; 67(6): 361–370. 6880820 10.1111/j.1600-0447.1983.tb09716.x

[10] Zigmond A Snaith R . The HADS: hospital anxiety and depression scale. Windsor, Canada: NFER Nelson, 1994.

[11] Stern AF . The hospital anxiety and depression scale. Occup Med 2014; 64(5): 393–394. 10.1093/occmed/kqu024 25005549

[12] Turk DC Dworkin RH Trudeau JJ , et al. Validation of the hospital anxiety and epression scale in patients with acute low back pain. The J Pain 2015; 16(10): 1012–1021. 26208762 10.1016/j.jpain.2015.07.001

[13] Stevens JP . Applied multivariate statistics for the social sciences. Oxfordshire, UK: Routledge, 2012.

[14] Mills S Torrance N Smith BH . Identification and management of chronic pain in primary care: a review. Curr Psychiatry Reports 2016; 18(2): 22. 10.1007/s11920-015-0659-9 PMC4731442 26820898

[15] Breivik H Collett B Ventafridda V , et al. Survey of chronic pain in Europe: prevalence, impact on daily life, and treatment. Eur Journal Pain 2006; 10(4): 287–287. 10.1016/j.ejpain.2005.06.009 16095934

[16] Henry SG Bell RA Fenton JJ , et al. Communication about chronic pain and opioids in primary care: impact on patient and physician visit experience. Pain 2018; 159(2): 371–379. 29112009 10.1097/j.pain.0000000000001098 PMC5934342

[17] Street RL Jr . How clinician–patient communication contributes to health improvement: modeling pathways from talk to outcome. Patient Education Counseling 2013; 92(3): 286–291. 23746769 10.1016/j.pec.2013.05.004

[18] Jose J . A healthcare professional's understanding of the medication information-seeking behaviour of patients: significance in the digital era. Oxford University Press, 2020, pp. 299–300. 10.1111/ijpp.12655 32643795

[19] Health NIF Excellence C . Medicines optimisation in long-term pain. London, UK: KTT21.NICE, 2017.

[20] Delgadillo J Huey D Bennett H , et al. Case complexity as a guide for psychological treatment selection. J Consulting Clinical Psychology 2017; 85(9): 835–853. 10.1037/ccp0000231 28857592

[21] Scott MJ . Ensuring that the improving access to psychological therapies (IAPT) programme does what it says on the tin. Br J Clin Psychol 2021; 60(1): 38–41. 32803761 10.1111/bjc.12264 PMC7891596

[22] Geneen LJ Moore RA Clarke C , et al. Physical activity and exercise for chronic pain in adults: an overview of Cochrane Reviews. The Cochrane Database Systematic Reviews 2017; 4(4): CD011279. 10.1002/14651858.CD011279.pub3 PMC5461882 28436583

[23] Rosenman R Tennekoon V Hill LG . Measuring bias in self-reported data. Int J Behav Healthc Res 2011; 2(4): 320–332. 25383095 10.1504/IJBHR.2011.043414 PMC4224297

[24] Jensen MP Karoly P . Self-report scales and procedures for assessing pain in adults. Handbook of pain assessment: The Guilford Press, 2011, pp. 19–44.

[25] Andrews P Steultjens M Riskowski J . Chronic widespread pain prevalence in the general population: a systematic review. Eur J Pain 2018; 22(1): 5–18. 28815801 10.1002/ejp.1090

[26] Armstrong K Putt M Halbert CH , et al. Prior experiences of racial discrimination and racial differences in health care system distrust. Med Care 2013; 51(2): 144–150. 23222499 10.1097/MLR.0b013e31827310a1 PMC3552105

[27] Hayanga B Stafford M Bécares L . Ethnic inequalities in healthcare use and care quality among people with multiple long-term health conditions living in the united kingdom: a systematic review and narrative synthesis. Int J Environ Res Public Health 2021; 18(23): 12599. 34886325 10.3390/ijerph182312599 PMC8657263

